# Air pollution, general government public-health expenditures and income inequality: Empirical analysis based on the spatial Durbin model

**DOI:** 10.1371/journal.pone.0240053

**Published:** 2020-10-01

**Authors:** Jianli Wu, Yue Pu

**Affiliations:** 1 Institute of Chinese Financial Studies, Southwestern University of Finance and Economics, Chengdu, Sichuan, China; 2 School of International Business, Southwestern University of Finance and Economics, Chengdu, Sichuan, China; The Bucharest University of Economic Studies, ROMANIA

## Abstract

Environmental pollution and income inequality are important issues related to sustainable economic and social development. Air pollution affects residents' physical health, and income inequality affects social stability and economic development. No scholar has yet confirmed the causal impact of air pollution on income inequality; therefore, this study is an important extension of the environmental Kuznets curve theory. This article examines the impact using balanced panel data from 156 countries (2004–2017) and applies the spatial Durbin model to analyze the mechanism of air pollution's impact on income inequality from the perspective of public health. The results prove the following. First, increasing air pollution does increase income inequality. Second, the spatial spillover effect of air pollution constitutes a relatively important part of the total effect of air pollution on income inequality compared with the direct effect. Third, general government public-health expenditures are an important transmission channel by which air pollution affects income inequality. The conclusions of the research have some important policy implications for environmental governance and income distribution policies at the national as well as supranational level.

## 1. Introduction

The theoretical research on the environment and income mainly involves the environmental Kuznets curve theory [1–3]. The theory was developed based on the observation of an inverted U-shaped relationship between economic growth and income inequality. This inverted U-shaped relationship between economic growth and income inequality was called the "Kuznets curve" [4]. The environmental Kuznets curve (EKC) theory states that there is also an inverted U-shaped relationship between per capita income growth and environmental degradation, but empirical research on EKC theory mainly focuses on the relationship between per capita income growth and environmental pollution or the relationship between income inequality and environmental pollution [5–15]. Few studies have taken the perspective of the impact of environmental pollution on income inequality. Therefore, this study provides an important extension for the existing research on the EKC theory.

Regarding the research on the relationship between income inequality and environmental pollution, most scholars have examined whether there is an inverted U-shaped relationship between income inequality and environmental quality and the impact of income inequality on environmental pollution. They believe that the expansion of income inequality leads to worse environmental pollution [5, 16–20]. However, early scholars discussed the impact of environmental pollution on income inequality. Brajer [21] used Chinese urban data (1995–2004) to measure three types of income inequality indicators for Chinese cities (Gini, Theil's T, and Theil's L index). Then, the author calculated the income inequality indicator between cities after adjusting for pollution. By comparing the changes in the income inequality index before and after the adjustment and examining the significance of the coefficients, the author concluded that the income inequality index adjusted by pollution has increased and is significant and therefore environmental pollution has widened the income inequality between cities. Slottje et al. [22] empirically tested the relationship between income disparity and environmental quality using US time-series data (1947–1996) and a vector error correction model. The authors observed the response of income inequality in different periods by assessing the standard error of the pollution variable. From an analysis of the results of the impulse response, the authors believed that an increase in pollution would lead to a decline in income inequality. That is, there is a negative relationship between income inequality and environmental pollution. In summary, we can find that the existing research not only does not outline a causal relationship between environmental pollution and income inequality, but it also does not reach a consistent conclusion. Therefore, the importance of this study lies in solving these two problems and providing constructive enlightenment for national economic and environmental governance.

**Fig 1:**
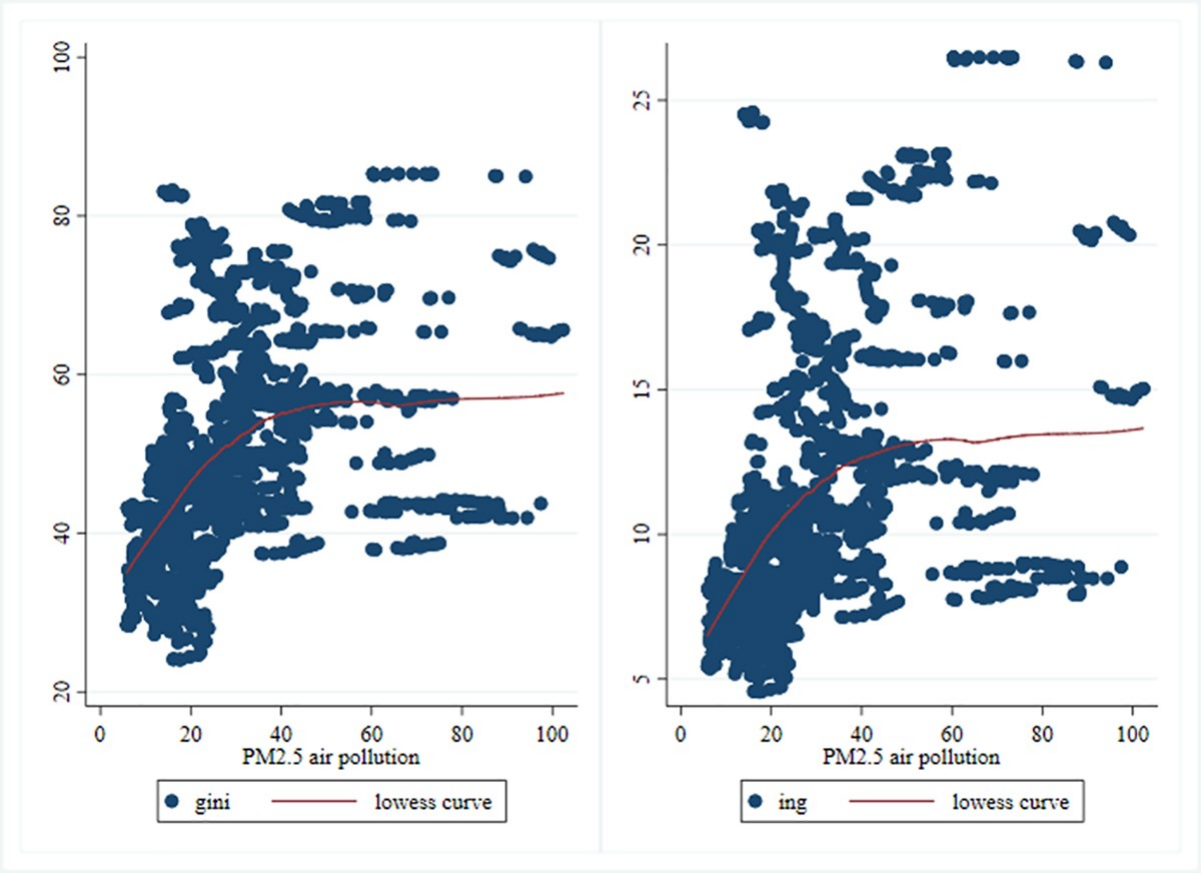
Scatter plot of air pollution and income inequality. The sample period is 2004–2017. "*gini*" and "*ing*" represent the Gini coefficient and the standard deviation of the income distribution data in each country from International Labour Organization Database (ILO), respectively, and are used as the references for the income inequality of each country [25]. "*PM2*.*5 air pollution*" represents the air pollution index of each country, and we use the annual average of each country’s PM2.5 exposure from Development Indicators Database (WDI) as a proxy indicator of air pollution. The "*lowess curve*" indicates the lowess line.

Fig 1 shows the scatter plot of air pollution and income inequality in 156 countries or regions (hereinafter collectively referred to as countries). As shown in Fig 1, there is a clear positive correlation between air pollution and income inequality [20, 23]. Although this differs from most previous research conclusions [16, 22, 24], it provides evidence for the impact of air pollution on income inequality.

The goal of this article is to examine the impact of air pollution on income inequality from a global perspective [22]. The study includes the following. First, the research hypotheses of this article are proposed based on the literature review to lay the foundation for the subsequent empirical analysis. Second, according to the compiled panel data for 156 countries (2004–2017), we empirically validate the validity of the research hypotheses and use the spatial Durbin model (SDM) to further investigate the spatial spillover effect of air pollution [26] and finally obtain consistent conclusions regarding the effect of air pollution on income inequality. Third, according to the transmission channel by which air pollution affects income inequality proposed in this article (air pollution-general government public-health expenditures-income inequality), the traditional mechanism verification method ("three-step method") is used to test the validity of this impact mechanism [27].

The innovations of the research work are as follows. First, most research on environmental pollution and income inequality have focused on the relationship between income inequality and environmental quality or environmental pollution [5, 19, 20]. Few studies have changed the research perspective to consider the impact of environmental pollution on income inequality. Because environmental pollution affects human health and residents' increasing medical expenditures on physical health increase income inequality, environmental pollution may affect income inequality. Therefore, this article provides a new perspective and an important extension for the EKC theory. Second, we use a spatial econometric method to determine the impact of air pollution on income inequality, which can intuitively reflect the spatial spillover effect on air pollution between countries. This method could avoid endogeneity problems to some extent due to omitted variables being one of the three reasons for endogeneity problems (omitted variables, measurement errors, and reverse causality). Third, this paper proposes an impact mechanism of air pollution on income inequality (air pollution-general government public-health expenditures-income inequality) and verifies it. Fourth, the conclusion that air pollution affects income inequality provides important policy implications for the sustainable development of the global environment, economy, and society.

The structure of the remainder of this article is as follows. The first part reviews the literature on environmental pollution, health, and income inequality and proposes the research hypotheses based on the existing research. The second part presents the model setting and the descriptions of the data and variables in this article. It lays the basis for the following empirical analysis by building a benchmark model. The third part is empirical analysis. This section includes the analysis of the benchmark regression results and the robustness tests and finally draws the empirical conclusions of the article. Then, we empirically verify the transmission mechanism (air pollution-general government public-health expenditures-income inequality) proposed in this article. Finally, the conclusions and policy implications of this article are presented.

## 2. Literature review

In this section, we mainly review two streams of literature: one stream addresses income inequality and environmental pollution, and the other addresses environmental pollution and health. We summarize the two research hypotheses in this paper by reviewing these two streams of the research literature and establish a framework for the subsequent empirical research.

### 2.1 Literature on income inequality and environmental pollution

The theoretical research on environmental pollution and income mainly originates from the EKC theory. Later studies carried out detailed analyses of the relationship between greenhouse gas emissions and income, then developed another theory, the environmental Engel curve (EEC) theory [1–3, 23, 28, 29]. Before the emergence of the EKC theory, scholars proposed that people treat environmental protection differently with income levels [30]. Later, scholars examined the relationship between per capita income and environmental quality by using actual data. They agreed that there is an inverted U-shaped relationship similar to the Kuznets curve between per capita income and environmental pollution [2, 3, 31]. This inverted U-shaped relationship indicates that as the level of per capita income increases, environmental pollution first deteriorates and then improves. The level of per capita income at the vertex is approximately $8,000 [2]. However, Selden and Song also pointed out that with the increase in per capita income, environmental improvement may occur for a long time. Regarding the economy, EKC theory mainly believes that in the early stage of economic growth, the environmental pressure on society increases faster than income; and after income reaches a high level, the environmental pressure on society increases more slowly than income [8]. However, some studies believe that the reason for the inverted U-shaped relationship between per capita income and environmental pollution is that the economy has scale effects, structural effects, and innovation effects.

Other research further investigated the impact of income inequality on environmental pollution based on the EKC theory. They confirmed that income inequality affects environmental pollution. Heerink et al. [24] used countries' Gini coefficients to empirically verify that widening income inequality improves the environmental quality [32]. However, Hao et al. [20] empirically examined the impact of income inequality on per capita carbon emissions using sample data for China and the Generalized method of moments (GMM) estimation. The results showed that as income inequality widens, an increase in per capita carbon emissions will result.

In summary, the existing theoretical and empirical studies have confirmed the impact of income inequality on environmental pollution, and some scholars have initially investigated the impact of environmental quality on income inequality [21, 22]. Therefore, we can formulate a research hypothesis about the impact of air pollution on income inequality based on the EKC theory:

**Hypothesis 1:** *As air pollution worsens*, *income inequality will further widen*.

### 2.2 Literature on environmental pollution and health

Since the beginning of the 21st century, environmental pollution has received increasing attention from governments and the general public. This is because environmental pollution not only affects the economy but also threatens human life and health [33]. The harm of air pollution to the human body is multifaceted and irreversible because air pollution can enter the body through the respiratory and digestive systems and cause serious damage to the human body [34–36]. The impact of air pollution on human health is mainly reflected in the following aspects. First, air pollution can cause skin damage, especially for people with congenital skin defects. Second, air pollution impacts the internal organs of the human body, such as the respiratory system, cardiovascular system, nervous system, urinary system, and digestive system [36–49].

Therefore, the increase in air pollution can cause different degrees of damage to various organs of the human body, which can lead to increasingly serious diseases affecting people's health. Health problems caused by air pollution further increase the health expenditures of households, businesses, and governments, and these increases can widen the income inequality between individuals [50–53]. For example, Lynch et al. [51] pointed out that compared with low-income groups, high-income groups obtain increasingly superior medical security and social benefits, which leads to increased life expectancy. In other words, health status is increasingly guaranteed as income increases. Therefore, we have reason to believe that there is a correlation between health problems caused by air pollution and the income inequality between the low-income and high-income groups [54].

Increasing air pollution leads to an increase in government expenditures on environmental governance and a corresponding reduction in general government public-health expenditures (transfer payments for low-income groups), and this is because high-income groups have better medical and welfare guarantees. In turn, the decline in general government public-health expenditures significantly increases the pressure on the medical expenditures of low-income groups; and as a result, the overall income inequality (between high-income and low-income groups) further widens. In summary, we can propose a hypothesis on the relationship between air pollution, general government public-health expenditures, and income inequality:

**Hypothesis 2:** *An increase in air pollution leads to a decrease in general government public-health expenditures*, *namely*, *a decline of transfer payments for the low-income groups*, *which in turn leads to widening the income inequality in society as a whole*.

## 3. Model and materials

### 3.1 Data

The sample data in this article are the balanced and annual panel data from 156 countries (2004–2017) in the world because the spatial econometric method requires a strongly balanced panel. Therefore, we performed trend interpolation on the missing values in the data. The data of the labor income distribution from the *International Labour Organization Database* (ILO) have been used to calculate the index of income inequality, and other explanatory variables and control variables are from the *World Bank's World Development Indicators Database* (WDI). Since the data on the income distribution of each country in the ILO database are only from 2004 to 2017, we limit the sample period of this article to 2004–2017. In addition, since the data of the two databases only match for 156 countries, we limit the number of countries in this article to 156.

### 3.2 Model

This paper uses a spatial econometric method to examine the impact of air pollution on income inequality. Therefore, we need to verify whether our sample data is suitable for using spatial econometric methods through statistical tests, and the specific tests and analysis are as the following.

**Fig 2:**
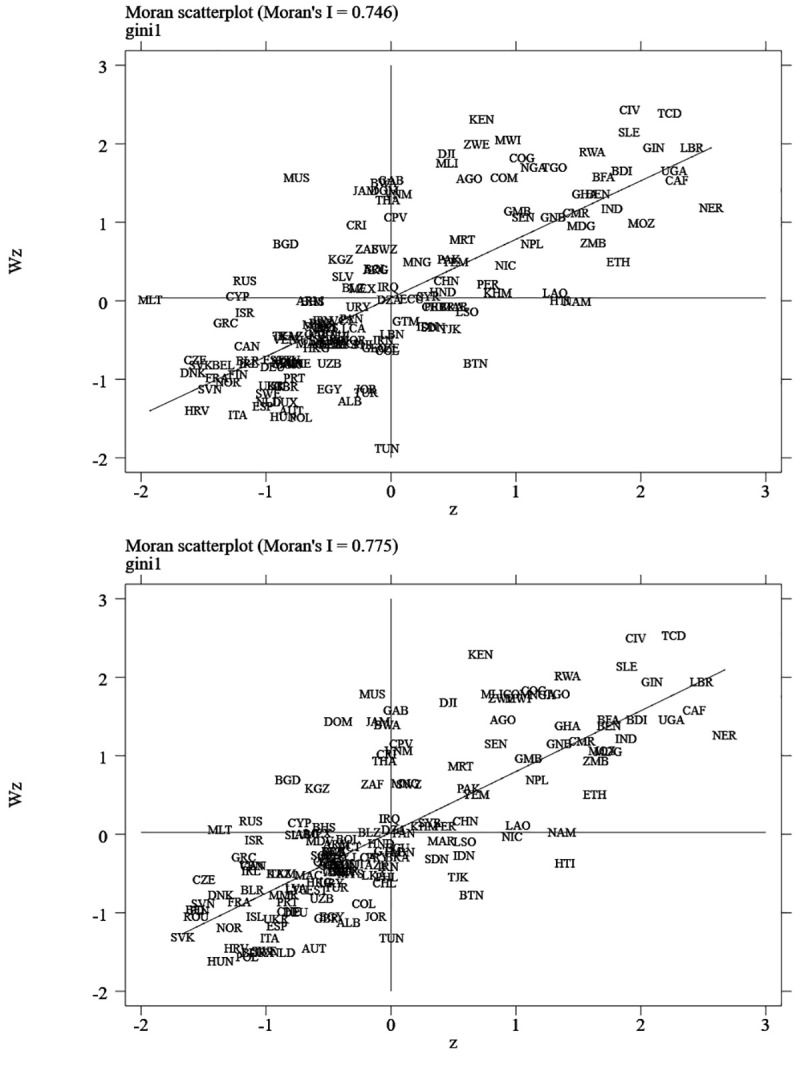
Moran quadrant plot of the spatial weight *wenco* (2004 and 2017). Each scatter point in the figures is marked by the ISO (International Standard Organization) code of each country. *Wz* denotes variable z’s spatial lag, and the four quadrants are HH, LH, LL, and HL, respectively. The scattered points are mostly located in quadrants one or three, indicating the phenomenon of spatial agglomeration. The same applies below.

First, we need to determine whether there are spatial effects of the research questions in this article. The test of this part is illustrated using a table and one figure (Fig 2 and Table 1). From the Moran scatter plots of the income inequality in Fig 2, it can be found that income inequality has a significant spatial agglomeration effect at the beginning of the sample period (2004) and at the end of the sample period (2017). In addition, under the economic distance matrix, the Moran is mainly concentrated in the first and third quadrants (the figures of the other three spatial weight matrices are given in S3–S5 Figs). Therefore, according to the Moran distribution in Fig 2, there is a positive spatial correlation of income inequality. From Table 1, it can be found that for the two types of spatial weight matrices of the geographic distance matrix (Euclidean distance), inverse geographic distance matrix, economic distance matrix (similarity of economic level), and inverse economic distance matrix, the Moran of air pollution is significant at the 1% significance level. In addition, during the period from 2004–2017, the Moran of air pollution remained highly significant. In summary, we can judge that the data in this paper have significant spatial effects, and we should use spatial econometric methods to analyze the research issues in this paper.

**Table 1:** Moran’s index over the years.

Variable	*I*	*z*	p-value*	*I*	*z*	p-value*	*I*	*z*	p-value*	*I*	*z*	p-value*
	*wbin*			*winv*			*wenco*			*weinv*		
2004	0.022	6.534	0.000	0.429	10.694	0.000	0.705	7.554	0.000	0.708	7.576	0.000
2005	0.023	6.802	0.000	0.430	10.724	0.000	0.708	7.588	0.000	0.711	7.609	0.000
2006	0.017	5.454	0.000	0.411	10.298	0.000	0.664	7.147	0.000	0.667	7.181	0.000
2007	0.019	5.853	0.000	0.414	10.383	0.000	0.669	7.199	0.000	0.672	7.229	0.000
2008	0.021	6.403	0.000	0.418	10.459	0.000	0.676	7.269	0.000	0.679	7.299	0.000
2009	0.023	6.874	0.000	0.421	10.523	0.000	0.683	7.336	0.000	0.686	7.363	0.000
2010	0.025	7.269	0.000	0.423	10.583	0.000	0.689	7.405	0.000	0.692	7.430	0.000
2011	0.023	6.786	0.000	0.419	10.465	0.000	0.685	7.359	0.000	0.688	7.389	0.000
2012	0.026	7.596	0.000	0.452	11.265	0.000	0.724	7.759	0.000	0.727	7.786	0.000
2013	0.027	7.860	0.000	0.453	11.297	0.000	0.715	7.672	0.000	0.717	7.688	0.000
2014	0.032	8.890	0.000	0.443	11.072	0.000	0.707	7.587	0.000	0.707	7.581	0.000
2015	0.034	9.315	0.000	0.449	11.189	0.000	0.735	7.866	0.000	0.737	7.881	0.000
2016	0.034	9.391	0.000	0.444	11.072	0.000	0.734	7.859	0.000	0.735	7.868	0.000
2017	0.034	9.319	0.000	0.442	11.031	0.000	0.736	7.883	0.000	0.737	7.891	0.000

Second, following the confirmation of the existence of spatial effects, we need to determine which spatial econometric model to select. Table 2 shows the results of the LR, AIC (Akaike Information Criterion), and BIC (Bayesian Information Criterion) tests. We first use the LR test to determine whether the SDM model, spatial error model (SEM model), or spatial lag model (SAR model) is more suitable for the sample in this article [55]. Then, we also use the AIC and BIC tests to determine whether the SDM model or the generalized spatial autoregressive model (SAC model) is more suitable for this study. The null hypothesis of the LR test is that the SDM model will be more effective than the SEM or SAR model; and according to the AIC and BIC tests, the model with the smaller AIC and BIC is advocated. From Table 2, we can find that the results of the LR test under the two types of spatial weights significantly reject the null hypothesis at the 1% significance level, that is, we should choose the SDM model as the benchmark model. The results of the AIC and BIC tests imply that the AIC and BIC values of the SDM model, under the two types of spatial weights, are mostly smaller than the SAC model; therefore, so we should choose the SDM model. In summary, we choose the SDM model as the empirical model for this paper.

**Table 2:** Adaptation test of spatial metrology model.

Testing method	*wbin*	*winv*	*wenco*	*weinv*
LR (SDM vs. SAR)	117.10***	77.36***	77.05***	77.89***
	(0.00)	(0.00)	(0.00)	(0.00)
LR (SDM vs. SEM)	111.79***	76.15***	74.58***	75.25***
	(0.00)	(0.00)	(0.00)	(0.00)
AIC(SDM)	6892.315	6939.195	6923.855	6921.995
AIC(SAC)	6985.079	6996.253	6958.299	6981.839
BIC(SDM)	7017.471	7064.351	7049.011	7047.151
BIC(SAC)	7059.035	7070.209	7032.255	7055.795

The standard errors are given in parentheses. Significance levels:

*** p < 0.01** p < 0.05, and * p < 0.1.

Finally, we need to choose and explain the parameter estimation methods for the empirical regression. The parameter estimation method in this paper is the individual-fixed effects method. The reasons for using the fixed effect method are as follows. First, for spatial panel data, if the random effect method is to be used, the number of observed sample points should approximately be infinity, however, this article does not satisfy this assumption. Second, for continuous national or regional spatial cross-sectional data or continuous space-time data, fixed effects are more suitable for estimating parameters than random effects. The sample data in this paper meet the requirements of a continuous space-time data structure. Third, we performed the Hausman test on the spatial panel data in this paper. Under the two types of spatial weight matrices, the test results significantly rejected the null hypothesis, that is, we should use the fixed effects method. Therefore, the parameter estimation method adopted in this paper is the individual-fixed effects method.

Before the empirical regression and analysis, we need to resolve the endogenous problem of air pollution. The endogeneity problem is inevitable in economic research. This problem refers to a case in which the explanatory variable is related to the error term. The existence of an endogeneity problem leads to inconsistency and bias of the estimation results. Therefore, to correctly estimate the effect of explanatory variables on the dependent variable, we need to find suitable instrumental variables instead of endogenous variables for parameter estimation. For this study, the endogeneity problem arises more from the reciprocal causation relationship between air pollution and income inequality according to the research on EKC theory.

Therefore, we need to find the appropriate instrumental variables for air pollution, but before looking for such variables, we need to explain several characteristics that the instrumental variables should have as follows. First, the instrumental variables must be related to the endogenous variable. Second, the instrumental variable must be unrelated to the disturbance term, which is also the condition for the exogenous instrumental variable. We have constructed two instrumental variables based on the data of each country’s latitude and imported automotive products. Following existing methods [56], we use the reciprocal of each country’s latitude multiplied by their total value of imported automotive products. Then, we take the third-order lag of the imported automotive products multiplied by the reciprocal latitude and the square of the third-order lag term as the instruments. We have some thoughts on the reasons for this as follows. First, using a world map, we can find that some developing countries with serious air pollution mostly are located in subtropical and tropical regions with lower latitudes. Therefore, there may be a negative relationship between the latitude and the air pollution. Second, an increase in imported vehicles can reduce domestic energy consumption in the production of automotive products and reduce air pollution. This is because the import of complete vehicles or parts of vehicles can increase the import of polluting products (e.g., steel parts) and reduce domestic air pollution [57]; therefore, the more automotive products were imported, the air pollution may be alleviated in the future. Third, since the latitude is a time-invariant variable, we multiply it by the time-varying variables (e.g., the imported automotive products), which is in line with the standard of the panel data structure. Finally, we use the third-order lag and square term because there are a time-lag effect and non-linear effects for the imported automotive products affecting air pollution. In addition, for the current period, the lagged variable is predetermined, and it might not be related to the disturbance term [56].

To test the validity of the instrumental variables in this paper, we also performed a two-stage least squares regression (2SLS) and some corresponding statistical tests. First, in the first-stage regression, the influence coefficients of the two instrumental variables on air pollution are significant at the 1% or 10% significance level, in which the sign of the coefficient of imported automotive products on air pollution is significantly negative (-0.163). In the second-stage regression results, the estimated parameters of air pollution on income inequality are significantly positive at the 1% significance level (0.907). Second, in addition, we also conduct an underrecognition test (Anderson canon. corr. LM test) and an overidentification test (Sargan test). The result of the underrecognition test is significant at the 5% significance level (7.938), and the outcome of the Sargan test is not statistically significant (5.548). This also verifies that our instrumental variables do not suffer from underrecognition and are exogenous on account of the null hypothesis of the overidentification test indicating that all instruments are exogenous.

Therefore, the empirical models of this study are set as follows:

$$\hat{pm25_{it}}=\gamma_{1}\cdot iv_{1}+\gamma_{2}\cdot iv_{2}+\kappa \cdot control_{it}+\nu_{it}$$ (1)

$$\begin{array}{l} gini_{it}=\rho \cdot W\cdot gini_{it}+\alpha_{0}\cdot {\hat{pm25}}_{it}+\alpha_{1}\cdot W\cdot {\hat{pm25}}_{it}+\beta \cdot control_{it}\\ \;+\delta \cdot W\cdot control_{it}+\xi_{i}+\varepsilon_{it} \end{array}$$ (2)

where *gini*_*it*_ indicates the income inequality of country *i* in year *t*; *pm25*_*it*_ indicates the air pollution of country *i* in year *t* (average annual PM2.5 exposure); *W* indicates the spatial weight matrix; *Wy*, *Wx* indicate endogenous and exogenous interaction effects, respectively; the direct and indirect effect is diagonal elements, off-diagonal elements of (*I−ρW*)^−1^(*α*_0_+*Wα*_1_), respectively; and *control*_*it*_ indicates the control variable of country *i* in year *t*, which affects the country's income inequality. In addition, *ξ*_*i*_ represents individual-fixed effects. In this paper, we construct two types of spatial weight matrices: a geographic distance matrix, an inverse geographic distance matrix, an economic distance matrix, and an inverse economic distance matrix.

### 3.3 Variable description

The dependent variable in this article is income inequality. We use the labour income distribution data of each country in the ILO database and apply the Gini coefficient and variance measurement formulas to compute proxy indicators of the two types of income inequality—the Gini coefficient and the standard deviation of the overall income distribution (*ing*) [58, 59] (pp.508-527). An increase in these two types of indicators indicates widening income inequality, and a decline indicates narrowing income inequality. The specific calculation formulas are as follows:

$$gini=\frac{1}{n}\sum_{i=2}^{n}\sum_{1=j<i}^{n}\left(p_{i}-p_{j}\right)$$ (3)

$$ing=\sqrt{\sum_{i}{\left(p_{i}-\bar{p}\right)}^{2}/n}$$ (4)

In formula (3) and formula (4), the total population of a country is divided into *n* equal parts, and *p*_*i*_ represents the proportion of the i-th population income in the total income (i-th decile).

From S1 Table, we can find the dynamic changes in income inequality and the distribution characteristics of income inequality across 156 countries (2004–2017). In terms of the changes in income inequality from 2004 to 2017, developed countries in Europe experienced a decline in income inequality, and countries in Southeast Asia, Central Asia, and Latin America (for example, Mexico, Brazil, and Argentina) also showed improved income inequality to varying degrees.

The independent variables in this article include air pollution (*pm25*), and we use the average annual PM2.5 exposure in each country as a proxy. An increase in the value of the indicator indicates worsening air pollution, and a decrease indicates improving air pollution. The direction of the impact of air pollution on income inequality has been explained in our research hypotheses and will not be repeated here. The proxy of aggregate output (*gdp*) is each country’s Gross Domestic Product—GDP. The growth of aggregate output has a positive or negative correlation with income inequality [4]. To measure government consumption (*gov*), we use the ratio of the total final consumption of each country's government as a proportion of its GDP as a proxy variable. The increase in government consumption indicates a corresponding increase in the scale of government transfer payments, which leads to a decline in the overall income inequality [60]. We use the dependency ratio of the working-age population to characterize the dependency ratio (*depen*). The dependency ratio indicates the population age distribution. The income inequality will increase when relatively large cohorts are young or old because relatively large cohorts likely obtain a low salary [61]. Thus, the higher dependency ratio has an association with higher income inequality. We measure foreign direct investment (*fdi*) using the proportion of net foreign investment in a country as a proportion of the total value of GDP. An increase in FDI will increase the income level of domestic residents and help reduce income inequality. However, this will also lead to widening regional income inequality caused by regional differences in foreign direct investment [62, 63]. We also consider the investment rate (*invr*) using the capital formation rate of each country as a proxy. The impact of the investment rate on income inequality is similar to the impact of foreign direct investment, with both positive and negative effects [64]. We characterize trade openness (*to*) by the proportion of total trade to GDP. Trade openness is conducive to increasing the income of domestic residents, which promotes the narrowing of income inequality [65]. We proxy for the degree of financial development (*fd*) using the net lending by financial institutions as a proportion of GDP. An increase in the level of financial development can reduce the financing cost of residents, which is conducive to increasing income and narrowing income inequality [66]. The urbanization rate (*tpr*) is represented by the proportion of the urban population to the total population. An increase in the urbanization rate is conducive to increasing the income of the new urban population and then reducing income inequality [67]. Population growth (*pg*) is measured by the population growth rate of each country. Increasing population growth is conducive to optimizing the age structure of the population, promoting economic growth, and reducing income inequality [68].

## 4. Results

### 4.1 Descriptive statistics

Table 3 and Table 4 describe the structural characteristics and correlation of the sample data, respectively. It can be seen from Table 3 that the distributions of each variable are well-behaved, and the sample data in each quantile maintain a steady change. In addition, the minimum values of the four variables *fdi*, *to*, *fd*, and *pg* are negative. *fdi* and *to* are negative because we have processed the natural logarithm of these two variables, and the sample points that are less than one become negative after we take the natural logarithm. *fd* and *pg* are negative because the net credit volume of a country's financial institutions and the population growth rate may be negative. Table 4 shows that the correlation coefficient between air pollution and income inequality is significantly positive at the 1% significance level, that is, there is a significant positive relation between air pollution and income inequality. The correlation coefficients between general government public-health expenditures, income inequality, and air pollution are significantly negative at the 1% significance level, that is, there is a negative relationship between changes in general government public-health expenditures and changes in income inequality or air pollution.

**Table 3:** Statistical description.

Variable	mean	sd	p5	p25	p50	p75	p95	min	max
*gini*	49.64	13.53	31.04	40.28	46.46	57.41	75.57	24.08	85.32
*ing*	11.01	4.54	6.00	7.85	9.55	12.98	20.56	4.55	26.49
*pm25*	29.24	17.75	8.98	17.15	25.18	35.50	69.15	5.86	102.37
*gghe*	3.29	2.18	0.70	1.61	2.75	4.45	7.62	0.15	14.54
*gdp*	4.18	15.27	0.02	0.10	0.41	2.35	18.57	0.01	194.85
*gov*	15.41	5.21	7.23	11.47	15.34	18.79	24.19	1.60	40.44
*depen*	4.04	0.31	3.60	3.86	3.98	4.27	4.56	2.76	4.72
*fdi*	-4.43	16.57	-15.35	-5.20	-2.12	-0.45	3.88	-265.92	157.39
*invr*	24.92	8.21	13.82	20.00	23.89	28.23	40.90	1.53	67.91
*to*	4.26	0.86	3.42	4.02	4.35	4.67	5.12	-1.79	6.09
*fd*	6.69	5.79	0.56	2.42	4.99	9.24	18.99	-2.79	31.66
*tpr*	3.97	0.47	2.95	3.71	4.08	4.35	4.54	2.21	4.61
*pg*	1.53	1.64	-0.45	0.55	1.34	2.38	3.63	-9.08	17.51

**Table 4:** Correlation coefficient matrix of each variable.

Variable	*gini*	*pm25*	*gghe*	*gdp*	*gov*	*depen*	*fdi*	*invr*	*to*	*fd*	*tpr*	*pg*
***gini***	1.000											
***pm25***	0.400***	1.000										
***gghe***	-0.602***	-0.547***	1.000									
***gdp***	-0.119***	-0.087***	0.368***	1.000								
***gov***	-0.321***	-0.254***	0.555***	0.068***	1.000							
***depen***	0.702***	0.132***	-0.324***	-0.131***	-0.105***	1.000						
***fdi***	-0.063***	0.032	0.073***	0.070***	0.001	-0.061***	1.000					
***invr***	0.002	0.204***	-0.148***	0.009	-0.003	-0.167***	-0.034	1.000				
***to***	-0.146***	-0.144***	0.168***	-0.126***	0.131***	-0.204***	-0.090***	0.097***	1.000			
***fd***	-0.503***	-0.338***	0.632***	0.285***	0.305***	-0.423***	0.038*	-0.067***	0.183***	1.000		
***tpr***	-0.625***	-0.338***	0.494***	0.176***	0.246***	-0.571***	0.038*	0.007	0.209***	0.438***	1.000	
***pg***	0.361***	0.385***	-0.328***	-0.125***	-0.185***	0.117***	0.038*	0.015	0.012	-0.217***	-0.116***	1.000

### 4.2 Benchmark results

Using the estimated parameters of the first stage of the 2SLS method, we have estimated an exogenous *pm25* using the instrumental variables and control variables. The benchmark regression results in this paper are shown in Table 5 using the maximum likelihood method for the SDM model. In addition, Table 5 shows the results using two types of spatial weight matrices (geographic distance, inverse geographic distance, economic distance matrix, and inverse economic distance matrices), and we take the first column as the benchmark.

**Table 5:** Results of spatial Durbin model.

Variable	(1)	(2)	(3)	(4)
	*wbin*	*winv*	*wenco*	*weinv*
*pm25*	0.630***	0.595***	0.722***	0.724***
	(0.157)	(0.162)	(0.161)	(0.161)
Control variable	Yes	Yes	Yes	Yes
***Wx***				
*pm25*	1.447*	0.988*	0.593***	0.593***
	(0.784)	(0.585)	(0.214)	(0.214)
Control variable	Yes	Yes	Yes	Yes
***Direct Effect***				
*pm25*	0.673***	0.646***	0.784***	0.785***
	(0.164)	(0.164)	(0.165)	(0.165)
Control variable	Yes	Yes	Yes	Yes
***Indirect Effect***				
*pm25*	4.060**	1.503**	0.774***	0.774***
	(1.829)	(0.723)	(0.222)	(0.221)
Control variable	Yes	Yes	Yes	Yes
***Total Effect***				
*pm25*	4.732**	2.150***	1.558***	1.559***
	(1.870)	(0.737)	(0.282)	(0.281)
*gdp*	0.128	-0.122*	0.011	0.010
	(0.138)	(0.069)	(0.014)	(0.014)
*gov*	0.335	-0.210***	-0.036	-0.040*
	(0.215)	(0.055)	(0.023)	(0.023)
*depen*	7.402**	2.621**	2.637***	2.559***
	(3.587)	(1.075)	(0.665)	(0.662)
*fdi*	-0.036	-0.013*	-0.002	-0.002
	(0.041)	(0.008)	(0.003)	(0.003)
*invr*	0.159**	0.062***	0.000	0.001
	(0.078)	(0.019)	(0.009)	(0.009)
*to*	2.607	0.118	-0.423*	-0.426*
	(1.795)	(0.536)	(0.256)	(0.257)
*fd*	-0.366	-0.035	-0.073***	-0.071***
	(0.234)	(0.059)	(0.027)	(0.027)
*tpr*	8.147	-0.199	-2.730***	-2.826***
	(6.184)	(1.888)	(1.003)	(0.997)
*pg*	0.235	-0.346***	-0.154***	-0.155***
	(0.561)	(0.090)	(0.049)	(0.049)
*ρ*	0.563***	0.273***	0.158***	0.158***
	(0.067)	(0.038)	(0.018)	(0.018)
*σ*^*2*^	1.386***	1.395***	1.409***	1.408***
	(0.042)	(0.042)	(0.043)	(0.043)
*Moran’s index*	9.580***	16.453***	11.989***	12.011***
*N*	2184	2184	2184	2184
*R*^*2*^	0.271	0.419	0.440	0.441
*Log-likelihood*	-3465.648	-3474.404	-3487.482	-3486.112
*Hausman test*	194.89***	99.65***	61.30**	68.23***

1. Because the table is too long, we did not show the results of the control variables. If reviewers and readers need it, we can provide complete results. 2. Computing marginal effects’ standard errors are adopted by Monte Carlo (MC) simulation.

From the effect decomposition results in Table 5, the following can be seen. First, the total effect (the completed marginal impacts) of the impact of air pollution on income inequality is positive at the 1% or 5% significance level; therefore, we can say that the worsening of air pollution will lead to the expansion of income inequality, and Hypothesis 1 is supported. That is, air pollution has a significantly positive impact on income inequality. Compared with the results of 2SLS, we can find if we apply a common panel data model, we will seriously underestimate the impact of air pollution on income inequality.

Second, the parameters regarding the direct effect of air pollution on income inequality are all significantly positive at the 1% significance level, and the impact directions are also consistent. The indirect effect (spatial spillover effects) of air pollution on income inequality are all positive at the 1% or 5% significance level, indicating that there is a significant spatial spillover effect of air pollution on income inequality. Finally, under the geographic distance matrix, the indirect effect of air pollution on income inequality accounts for 85.80% of the total effect. For the last two spatial weight matrices, the proportion of indirect effect to the total effect is smaller than 50%. From the above analysis, it can be seen that the indirect effect is the main component of the total effect.

In addition, the impact coefficient of lagged income inequality on domestic income inequality is *ρ*. The results in Table 5 show that *ρ* is significant at the 1% significance level in all spatial weight matrices.

### 4.3 Robustness test

Although we have drawn benchmark results of the relationship between air pollution and income inequality, we still need to further test whether this relationship is affected by different empirical methods and indicators. Therefore, we have conducted two main tests of the robustness as the following.

#### Robustness of the method

We have used the fixed effect method to regress the benchmark outcomes considering that this approach can control the omitted variables of time-invariant to obtain the consistent estimator; however, in the Bayesian analysis framework, the reason why we use the fixed effects might be no longer supported. Therefore, we have added this section of the Bayesian analysis framework applying random effects to support a robustness proof of the above conclusions. In the section, we have applied the MCMC and INLA methods [69–72], and the main outcomes are listed in Table 6. In addition, the diagnostic results of the MCMC method do not have an obvious problem, as shown in S2 Fig. In Table 6, we provide three outcomes using the MCMC and INLA methods, respectively, and the last two columns applied the INLA method using panel data and spatial data (applying the geographic distance matrix), respectively. We find that the impact coefficient of air pollution on income inequality is still statistically significant and positive. That is, even if applying the Bayesian analysis approach, we can always achieve consistent results with the benchmark results. In addition, most of the control variables are also statistically significant.

**Table 6:** MCMC and INLA analysis.

Variable	Mean	Sd	Mean	Sd	Mean	Sd
	*MCMC*		*INLA_panel*		*INLA_spatial*	
*pm25*	0.915	0.058	0.808	0.164	0.953	0.190
*gdp*	0.024	0.003	0.025	0.009	0.019	0.010
*gov*	-0.027	0.006	-0.028	0.016	-0.036	0.018
*depen*	2.249	0.049	2.502	0.493	2.124	0.577
*fdi*	0.000	0.001	0.001	0.002	0.000	0.003
*invr*	-0.008	0.003	-0.006	0.006	-0.008	0.007
*to*	-0.030	0.033	-0.036	0.114	-0.008	0.133
*fd*	-0.057	0.005	-0.059	0.018	-0.038	0.022
*tpr*	-4.511	0.055	-5.744	0.737	-4.384	0.908
*pg*	-0.079	0.016	-0.075	0.034	-0.074	0.040
*Intercept*	69.995	0.145	63.747	4.161	87.319	6.491
*N*	2184		2184		2184	

#### Robustness of the indicator

In addition to the benchmark regression, we use another indicator of income inequality (*ing*)—the standard deviation of the income distribution in each country—as a robustness test for this article. We still use the construction method of formula (2):

$$\begin{array}{l} ing_{it}=\rho \cdot W\cdot ing_{it}+\alpha_{0}\cdot {\hat{pm25}}_{it}+\alpha_{1}\cdot W\cdot {\hat{pm25}}_{it}+\beta \cdot control_{it}\\ \;+\delta \cdot W\cdot control_{it}+\xi_{i}+\varepsilon_{it} \end{array}$$ (5)

In Table 7, we can find that, first, the directions of both direct effect and indirect effect of air pollution on income inequality are still consistent under the two types of spatial weight matrices, and the coefficients of the direct and indirect effect are all statistically significant and positive. Second, the influence directions of the direct effects of air pollution on income inequality under the two types of spatial matrices are consistent, and the total effects are all significantly positive at the 1% or 5% significance level. Finally, the spatial effect (*ρ*) of foreign income inequality on domestic income inequality are all significantly positive under all spatial matrices. Overall, the results in this section are still consistent with the benchmark results.

**Table 7:** Regression results of the second income inequality indicator (*ing*).

Variable	(1)	(2)	(3)	(4)
	*wbin*	*winv*	*wenco*	*weinv*
*pm25*	0.170***	0.167***	0.198***	0.198***
	(0.043)	(0.044)	(0.044)	(0.044)
Control variable	Yes	Yes	Yes	Yes
***Wx***				
*pm25*	0.359*	0.213	0.200***	0.195***
	(0.214)	(0.160)	(0.059)	(0.059)
Control variable	Yes	Yes	Yes	Yes
*ρ*	0.569***	0.255***	0.151***	0.150***
	(0.066)	(0.037)	(0.018)	(0.018)
*σ*^*2*^	0.103***	0.104***	0.106***	0.106***
	(0.003)	(0.003)	(0.003)	(0.003)
***Direct Effect***				
*pm25*	0.181***	0.178***	0.217***	0.216***
	(0.045)	(0.045)	(0.045)	(0.045)
Control variable	Yes	Yes	Yes	Yes
***Indirect Effect***				
*pm25*	1.040**	0.325*	0.250***	0.245***
	(0.498)	(0.193)	(0.060)	(0.060)
Control variable	Yes	Yes	Yes	Yes
***Total Effect***				
*pm25*	1.221**	0.503**	0.467***	0.461***
	(0.510)	(0.197)	(0.077)	(0.076)
Control variable	Yes	Yes	Yes	Yes
*N*	2184	2184	2184	2184
*R*^*2*^	0.353	0.509	0.492	0.492
*Log-likelihood*	-625.056	-634.016	-658.545	-657.657

### 4.4 Mechanism verification

In the previous section, we analyzed the causal relationship of the effect of air pollution on income inequality. In this section, we further verified the transmission channel (intermediary variable, namely, general government public-health expenditures) by which air pollution affects income inequality, namely, air pollution-general government public-health expenditures-income inequality. In the analysis of Hypothesis 2, we have stated that increasing air pollution in a country will increase the government expenditures for improving the environment, which might cause the government to reduce the scale of general government public-health expenditures (transfer payments for low-income groups). The decreasing general government public-health expenditures may significantly reduce the medical benefits enjoyed by low-income groups, and the worsening air pollution likely leads to a higher incidence of health problems, which will cause low-income groups to pay a larger proportion of their income to maintain their health [46, 47, 49]. Nevertheless, high-income groups can enjoy better medical security and social benefits than low-income groups, and even worsening air pollution will not cause a significant increase in their private health expenditures [51, 73]. This, in turn, widens the country's overall income inequality. In addition, the scatter plot between income inequality and general government public-health expenditures can be seen in S1 Fig.

In this section, we use the traditional three-step method to verify the transmission channels proposed in this paper [27]. The reason why the Sobel, Bootstrap, and other methods are not used to verify the transmission channels in this paper is because the benchmark model of this paper does not apply these methods [74–76]. In addition, in the benchmark regression, we verified the first step of the three-step method using the SDM model. Next, following the pattern, we will still test the validity of the second and third steps using the SDM model. The specific models are set as follows:

$$\begin{array}{l} gghe_{it}=\rho \cdot W\cdot gghe_{it}+\alpha_{0}\cdot {\hat{pm25}}_{it}+\alpha_{1}\cdot W\cdot {\hat{pm25}}_{it}+\beta \cdot control_{it}\\ \;+\delta \cdot W\cdot control_{it}+\xi_{i}+\varepsilon_{it} \end{array}$$ (6)

$$\begin{array}{l} gini_{it}=\rho \cdot W\cdot gini_{it}+\alpha_{0}\cdot {\hat{pm25}}_{it}+\alpha_{1}\cdot W\cdot {\hat{pm25}}_{it}+\alpha_{2}\cdot gghe_{it}+\alpha_{3}\cdot W\cdot gghe_{it}\\ \;+\beta \cdot control_{it}+\delta \cdot W\cdot control_{it}+\xi_{i}+\varepsilon_{it} \end{array}$$ (7)

where *gghe*_*it*_ indicates the general government public-health expenditures of country *i* in year *t*, and the definitions of other variables and parameters are the same as in Eq (2).

In this section, we only take the significance of coefficients of the total effect as the criterion determining whether the transmission mechanism is supported in this study. From Table 8, we can see that the total effects of air pollution on general government public-health expenditures are negative at the 1% or 10% significance level, that is, increasing air pollution leads to lower general government health spending. Although the direct effects of air pollution on general government public-health expenditures are positive, the indirect effects are all negative at the 1% or 10% significance level.

**Table 8:** Mechanism verification (*Second step*).

Variable	(1)	(2)	(3)	(4)
	*wbin*	*winv*	*wenco*	*weinv*
*pm25*	0.001	0.026***	0.023***	0.023***
	(0.005)	(0.006)	(0.006)	(0.006)
Control variable	Yes	Yes	Yes	Yes
***Wx***				
*pm25*	-0.019*	-0.074***	-0.048***	-0.048***
	(0.011)	(0.011)	(0.006)	(0.006)
Control variable	Yes	Yes	Yes	Yes
*ρ*	0.061	0.219***	0.122***	0.122***
	(0.101)	(0.039)	(0.017)	(0.017)
*σ*^*2*^	0.237***	0.232***	0.235***	0.235***
	(0.007)	(0.007)	(0.007)	(0.007)
***Direct Effect***				
*pm25*	0.001	0.024***	0.020***	0.021***
	(0.005)	(0.006)	(0.006)	(0.006)
Control variable	Yes	Yes	Yes	Yes
***Indirect Effect***				
*pm25*	-0.021*	-0.086***	-0.049***	-0.049***
	(0.011)	(0.012)	(0.006)	(0.006)
Control variable	Yes	Yes	Yes	Yes
***Total Effect***				
*pm25*	-0.020*	-0.062***	-0.028***	-0.029***
	(0.011)	(0.009)	(0.005)	(0.005)
Control variable	Yes	Yes	Yes	Yes
*N*	2184	2184	2184	2184
*R*^*2*^	0.074	0.371	0.430	0.427
*Log-likelihood*	-1525.578	-1510.462	-1527.910	-1527.467

From Table 9, it can be found that under all the spatial weight matrices, the total effects of air pollution on income inequality are significantly positive at the 1% or 10% significance level under all spatial weight matrices. The total effects of general government public-health expenditures on income inequality are all significantly negative under the two types of spatial weight matrices.

**Table 9:** Mechanism verification (*Third step*).

Variable	(1)	(2)	(3)	(4)
	*wbin*	*winv*	*wenco*	*weinv*
*pm25*	0.667***	0.621***	0.731***	0.726***
	(0.156)	(0.162)	(0.160)	(0.160)
*gghe*	-0.030	0.014	-0.054	-0.054
	(0.052)	(0.052)	(0.052)	(0.052)
Control variable	Yes	Yes	Yes	Yes
***Wx***				
*pm25*	2.060***	0.371	0.591***	0.593***
	(0.786)	(0.595)	(0.215)	(0.215)
*gghe*	-2.422***	-0.770***	-0.209***	-0.205***
	(0.385)	(0.144)	(0.052)	(0.052)
Control variable	Yes	Yes	Yes	Yes
*ρ*	0.346***	0.226***	0.150***	0.150***
	(0.089)	(0.040)	(0.018)	(0.018)
*σ*^*2*^	1.369***	1.382***	1.399***	1.398***
	(0.041)	(0.042)	(0.043)	(0.042)
***Direct Effect***				
*pm25*	0.696***	0.645***	0.791***	0.786***
	(0.161)	(0.163)	(0.163)	(0.163)
*gghe*	-0.056	-0.013	-0.072	-0.073
	(0.050)	(0.050)	(0.050)	(0.050)
Control variable	Yes	Yes	Yes	Yes
***Indirect Effect***				
*pm25*	3.590***	0.689	0.785***	0.786***
	(1.349)	(0.780)	(0.243)	(0.243)
*gghe*	-3.747***	-0.972***	-0.243***	-0.238***
	(0.603)	(0.183)	(0.056)	(0.056)
Control variable	Yes	Yes	Yes	Yes
***Total Effect***				
*pm25*	4.286***	1.334*	1.575***	1.572***
	(1.374)	(0.784)	(0.291)	(0.290)
*gghe*	-3.803***	-0.985***	-0.315***	-0.311***
	(0.612)	(0.192)	(0.078)	(0.077)
Control variable	Yes	Yes	Yes	Yes
*N*	2184	2184	2184	2184
*R*^*2*^	0.320	0.550	0.498	0.497
*Log-likelihood*	-3445.327	-3460.055	-3477.747	-3476.689

Here, we can consider the total effect of air pollution on general government public-health expenditures in Table 8 as coefficient *a*, the total effect of general government public-health expenditures on income inequality in Table 9 as coefficient *b*, and the total effect of air pollution on income inequality in Table 9 as coefficient *c'* (direct effect of the mediating effect). For the three-step method mechanism test, we mainly judge the joint significance of *ab* (indirect effect of the mediating effect). If both *a* and *b* are significant, it can be assumed that *ab* is also significant, that is, there is a mediating effect [77, 78]. Because *a* and *b* in Table 8 and Table 9 are all statistically significant, we can approximately consider that the impact mechanism of this article is valid, and Hypothesis 2 is supported.

## 5. Discussion

This paper investigates the impact of air pollution on income inequality from the perspective of public health using balanced panel data from 156 countries (2004–2017) and applying the spatial Durbin model to analyze the mechanism. This study finds a causal relationship between air pollution and income inequality which implies that air pollution has a significantly positive impact on income inequality, and, this is an important extension of the environmental Kuznets curve theory.

This study used the spatial Durbin model to regress our outcomes and control the individual-fixed effects simultaneously. The reasons include the following, first, the research object (air pollution) of this study has obvious spillover effects and negative externality in reality and this fact means that air pollution may have a certain spatial interaction effect in empirical terms. Second, for the ordinary panel data model, the direct effect of the independent variable is equal to the estimated coefficient, while its indirect effect is zero by construction [79]. This will cause us to be unable to identify all the effects and result in inconsistent estimators of the explanatory variable on the explained variable. Third, Elhorst and Fréret [80] thought that controlled fixed effects for all space-specific, time-invariant variables could prevent biasing the estimates in a typical cross-sectional study. In addition, controlling the omissions could also mitigate the endogeneity problems of the omitted variables to some extent.

For the analysis of the outcomes of spatial econometric models, our main concern is the significance of the direct effect, indirect effect (the spatial spillovers), and total effect (the completed marginal impacts) coefficients. The direct effect indicates that changes of a particular explanatory variable in a particular unit will result in the dependent variable in that unit to change. The indirect effect indicates that changes of a particular explanatory variable in a particular unit will result in the dependent variables in other units to change [79, 81, 82]. And, the total effect is the total marginal impact of explanatory variables on the dependent variable [79, 82].

Furthermore, if the coefficients of *x*, *Wx*, and *Wy* in the spatial Durbin model happen to be not significant, this does not automatically represent that the indirect effect of the explanatory variable is not statistically significant [79].

Therefore, the results of this study become more credible than those of Brajer [21] and Slottje et al. [22], since they just applied common qualitative and quantitative methods without considering the endogeneity problems and the spatial spillover effects. And, Brajer [21] and Slottje et al. [22] have some contradictory conclusions. This paper aims to verify the consistent causal relationship between air pollution and income inequality based on their works. Compared with existing research, this study added the spatial spillover effects and applied the IV method. We need to construct an instrument for the endogenous variable (air pollution), then we replace *Y = (I-ρW)*^*-1*^*(βX+θWX)+ε* with *Y = (I-ρW)*^*-1*^*(βZ+θWZ)+ε* in which *Z* indicates the first-step estimator of 2SLS on air pollution using instrumental variables. In reality, we used the estimated parameters of the first stage of 2SLS method rather than using spatial IV/GMM to resolve the endogeneity of air pollution [83–85]. These mainly include two reasons. First, the present spatial IV/GMM method is not suitable for the spatial Durbin model with fixed-effect, and the endogeneity does not come from spillovers. Second, one disadvantage of the IV/GMM estimator is the possibility of ending up with a coefficient estimate outside its parameter space [79].

From the empirical outcomes, the increasing air pollution in neighboring countries has a spatial spillover effect or negative externality on the home country’s air [79, 80], which further worsens the domestic air pollution and in turn, increases the environmental governance costs of the home country. Afterward, domestic governments (given the scale of government expenditures) will cut expenditures for other government public projects (e.g., the general government public-health expenditures meaning transfer payments for low-income groups), and these might lead to the changes of the income inequality. Among them, an analysis of how an increase on air pollution in the home country widens domestic income inequality is presented in Hypothesis 2. The above analysis method is similar to Elhorst [79]. For example, Elhorst [79] thought that the changes in one state's price level or income level would affect not only the consumption of cigarettes in the home state but also the consumption of cigarettes in neighboring states (namely, a feedback effect). The viewpoints were not consistent with Baltagi and Levin [86]. From the above analysis, we can clearly find that if we take the effects of domestic air pollution on domestic income inequality as the benchmark result, we will generally underestimate the impact of air pollution on income inequality [22]. Therefore, such a conclusion is obviously inaccurate, and it also provides evidence for the rationality of why the spatial econometric method using IV variables is applied to investigate the impact of air pollution on income inequality.

In addition, following the research experience of Barceló and Rue et al. [69–72], we have also retested the empirical outcomes with Bayesian analysis methods (e.g., the MCMC model and the INLA model). Bayesian analysis methods are used because this study used the fixed-effects method to regress, and we should use other methods as a comparison. Fortunately, the Bayesian analysis outcomes are in line with those of the spatial Durbin model.

The limitations of the study are as follows. First, the study did not provide a theoretical analysis of the impact of air pollution on income inequality. This might affect the completeness of economic research. In the future, we will supplement this section. Second, due to us not being masters of Bayesian analysis methods, in this paper, we just used some simple commands to get the outcomes. Through future systematic learning, we hope to expertly apply Bayesian analysis methods. Third, the study did not verify the mechanism channel using Sobel or Bootstrap methods. These might make this study’s mechanism test more credible. Therefore, we will improve these insufficiencies using other models in the future.

## 6. Conclusions

Based on a review of the literature, this paper proposes two research hypotheses regarding the impact of air pollution on income inequality using both balanced panel data from 156 countries (2004–2017) and a spatial Durbin model to empirically test the research hypotheses. We get the following findings. First, the total effect of air pollution on income inequality is significantly positive, that is, the more serious that air pollution is, the larger the income inequality becomes. Second, the impact of air pollution on income inequality is mainly through indirect effects (spatial spillover effects). Third, the transmission channel by which air pollution affects income inequality (air pollution-general government public-health expenditures-income inequality) is supported.

The main point of this article is that the worsening of air pollution leads to a widening of income inequality, and air pollution has a strong spatial spillover effect. These points have important implications for policymakers in every country. First, excessive income inequality is deemed to be harming for socio-political stability and economic growth [87, 88]. Therefore, a sound income distribution system plays an important role in reducing income inequality and promoting economic growth. Second, owing to air pollution harms health and affects residents’ lives and income, the policy of environmental governance is conducive to control air pollution and its “side effects”. Third, the traditional political economy models imply that in democracies the decisive voter is an individual with lower-than-average income [89]. Therefore, in democracies, the restrictive environmental governance policies may be easier to implement than what is typically believed. Finally, the spatial indirect effects (spatial spillover effects) of air pollution are large in magnitude, implying in turn that local and national governments in isolation may optimally choose a sub-optimally low level of environmental regulation. As a consequence, this strengthens the policy prescription that environmental regulation should be agreed upon and cooperated at a supranational level.

## Supporting information

S1 Table The change of gini index. (PDF)

S2 Table Variable description. (PDF)

S3 Table Country name and country code. (PDF)

S1 Fig Scatter plot of general government public-health expenditures and income inequality. (TIF)

S2 Fig Diagnostic chart of MCMC. (TIF)

S3 Fig Moran quadrant plot of the spatial weight *wbin* (2004 and 2017). (TIF)

S4 Fig Moran quadrant plot of the spatial weight *winv* (2004 and 2017). (TIF)

S5 Fig Moran quadrant plot of the spatial weight *weinv* (2004 and 2017). (TIF)

S1 File The codes and data. (ZIP)
