# Supplementary material for: Air pollution, general government public-health expenditures and income inequality: Empirical analysis based on the spatial Durbin model
Source: PLoS One. 2020 Oct 1;15(10):e0240053. doi: 10.1371/journal.pone.0240053 (PMC7529191; doi:10.1371/journal.pone.0240053)
Supplement: S1 Table — (PDF) [file pone.0240053.s001.pdf]

S1 Table. The change of gini index

Serial	Name	Code	Region	Δ(gini_2017-	Serial	Name	Code	Region	Δ(gini_2017-gini_2014)
Top20 countries					Last20 countries				
125	Romania	ROU	Europe & Central Asia	-10.624	7	Austria	AUT	Europe & Central Asia	1.227
82	Cambodia	KHM	East Asia & Pacific	-9.383	138	Eswatini	SWZ	Sub-Saharan Africa	1.262
4	Argentina	ARG	Latin America & Caribbean	-8.683	40	Germany	DEU	Europe & Central Asia	1.265
64	Honduras	HND	Latin America & Caribbean	-8.066	52	France	FRA	Europe & Central Asia	1.288
67	Hungary	HUN	Europe & Central Asia	-7.949	96	Madagascar	MDG	Sub-Saharan Africa	1.289
123	Paraguay	PRY	Latin America & Caribbean	-7.669	5	Armenia	ARM	Europe & Central Asia	1.615
20	Brazil	BRA	Latin America & Caribbean	-7.217	89	Sri Lanka	LKA	South Asia	1.624
121	Poland	POL	Europe & Central Asia	-7.016	42	Denmark	DNK	Europe & Central Asia	1.89
27	Chile	CHL	Latin America & Caribbean	-6.721	37	Costa Rica	CRI	Latin America & Caribbean	1.945
133	El Salvador	SLV	Latin America & Caribbean	-6.373	1	Angola	AGO	Sub-Saharan Africa	2.151
43	Dominican Republic	DOM	Latin America & Caribbean	-6.336	75	Italy	ITA	Europe & Central Asia	2.296
119	Peru	PER	Latin America & Caribbean	-6.141	68	Indonesia	IDN	East Asia & Pacific	2.529
98	Mexico	MEX	Latin America & Caribbean	-6.075	54	United Kingdom	GBR	Europe & Central Asia	3.14
103	Mozambique	MOZ	Sub-Saharan Africa	-5.961	65	Croatia	HRV	Europe & Central Asia	3.145
85	Lao PDR	LAO	East Asia & Pacific	-5.577	99	Mali	MLI	Sub-Saharan Africa	3.41
55	Georgia	GEO	Europe & Central Asia	-5.326	48	Estonia	EST	Europe & Central Asia	3.489
50	Finland	FIN	Europe & Central Asia	-5.29	118	Panama	PAN	Latin America & Caribbean	4.338
14	Bulgaria	BGR	Europe & Central Asia	-4.844	31	Cyprus	CYP	Europe & Central Asia	5.518
154	Venezuela, RB	VEN	Latin America & Caribbean	-4.83	100	Malta	MLT	Middle East & North Africa	6.484
147	Turkey	TUR	Europe & Central Asia	-4.496	105	Mauritius	MUS	Sub-Saharan Africa	6.949
