# Supplementary material for: Air pollution, general government public-health expenditures and income inequality: Empirical analysis based on the spatial Durbin model
Source: PLoS One. 2020 Oct 1;15(10):e0240053. doi: 10.1371/journal.pone.0240053 (PMC7529191; doi:10.1371/journal.pone.0240053)
Supplement: S2 Table — (PDF) [file pone.0240053.s002.pdf]

S2 Table. Variable description

Variable	Short name	Data sources
Income inequality	*gini*	ILO https://www.ilo.org/shinyapps/bulkexplorer12/?lang=en&segment=indicator&id=LAP_2LID_QTL_DT_A
	ing	
Air pollution	*pm25*	WDI https://datacatalog.worldbank.org/dataset/world-development-indicators
General government public-health expenditures	*gghe*	
Gross Domestic Product	*gdp*	
Government consumption	*gov*	
Dependency ratio	*depen*	
Foreign direct investment	*fdi*	
Investment rate	*invr*	
Trade openness	*to*	
Degree of financial development	*fd*	
Urbanization rate	*tpr*	
Population growth	*pg*	
Imported automotive products	*iv*	WTO https://timeseries.wto.org/
