# Supplementary material for: Air pollution, general government public-health expenditures and income inequality: Empirical analysis based on the spatial Durbin model
Source: PLoS One. 2020 Oct 1;15(10):e0240053. doi: 10.1371/journal.pone.0240053 (PMC7529191; doi:10.1371/journal.pone.0240053)
Supplement: S3 Table — (PDF) [file pone.0240053.s003.pdf]

S3 Table. Country name and country code

Serial	Code	Name	Serial	Code	Name	Serial	Code	Name	Serial	Code	Name
1	AGO	Angola	41	DOM	Dominica	81	KWT	Kuwait	121	QAT	Qatar
2	ALB	Albania	42	DZA	Algeria	82	LAO	Laos	122	ROU	Romania
3	ARE	United Arab Emirates	43	ECU	Ecuador	83	LBN	Lebanon	123	RUS	Russia
4	ARG	Argentina	44	EGY	Egypt	84	LBR	Liberia	124	RWA	Rwanda
5	ARM	Armenia	45	ESP	Spain	85	LCA	St. Lucia	125	SAU	Saudi Arabia
6	AUS	Australia	46	EST	Estonia	86	LKA	Sri Lanka	126	SDN	Sudan
7	AUT	Austria	47	ETH	Ethiopia	87	LSO	Lesotho	127	SEN	Senegal
8	AZE	Azerbaijan	48	FIN	Finland	88	LTU	Lithuania	128	SGP	Singapore
9	BDI	Burundi	49	FJI	Fiji	89	LUX	Luxembourg	129	SLE	Sierra Leone
10	BEL	Belgium	50	FRA	France	90	LVA	Latvia	130	SLV	El Salvador
11	BEN	Benin	51	GAB	Gabon	91	MAC	Macao, China	131	SUR	Suriname
12	BFA	Burkina Faso	52	GBR	United Kingdom	92	MAR	Morocco	132	SVK	Slovakia
13	BGD	Bangladesh	53	GEO	Georgia	93	MDG	Madagascar	133	SVN	Slovenia
14	BGR	Bulgaria	54	GHA	Ghana	94	MDV	Maldives	134	SWE	Sweden
15	BHR	Bahrain	55	GIN	Guinea	95	MEX	Mexico	135	SWZ	Isvetini
16	BHS	Bahamas	56	GMB	Gambia	96	MLI	Mali	136	SYR	Syria
17	BLR	Belarus	57	GNB	Guinea-Bissau	97	MLT	Malta	137	TCD	Chad
18	BLZ	Belize	58	GRC	Greece	98	MMR	Myanmar	138	TGO	Togo
19	BOL	Bolivia	59	GTM	Guatemala	99	MNG	Mongolia	139	THA	Thailand
20	BRA	Brazil	60	HKG	China Hong Kong	100	MOZ	Mozambique	140	TJK	Tajikistan
21	BRB	Barbados	61	HND	Honduras	101	MRT	Mauritania	141	TKM	Turkmenistan
22	BTN	Bhutan	62	HRV	Croatia	102	MUS	Mauritius	142	TTO	Trinidad and Tobago
23	BWA	Botswana	63	HTI	Haiti	103	MWI	Malawi	143	TUN	Tunisia
24	CAF	Central African Republic	64	HUN	Hungary	104	MYS	Malaysia	144	TUR	Turkey

25	CAN	Canada	65	IDN	Indonesia	105	NAM	Namibia	145	UGA	Uganda
26	CHE	Switzerland	66	IND	India	106	NER	Niger	146	UKR	Ukraine
27	CHL	Chile	67	IRL	Ireland	107	NGA	Nigeria	147	URY	Uruguay
28	CHN	China	68	IRN	Iran	108	NIC	Nicaragua	148	USA	United States
29	CIV	Ivory Coast	69	IRQ	Iraq	109	NLD	Netherlands	149	UZB	Uzbekistan
30	CMR	Cameroon	70	ISL	Iceland	110	NOR	Norway	150	VCT	Saint Vincent and the Grenadines
31	CYP	Cyprus	71	ISR	Israel	111	NPL	Nepal	151	VEN	Venezuela
32	COG	Republic of Congo)	72	ITA	Italy	112	NZL	New Zealand	152	VNM	Vietnam
33	COL	Colombia	73	JAM	Jamaica	113	OMN	Oman	153	YEM	Yemen
34	COM	Comoros	74	JOR	Jordan	114	PAK	Pakistan	154	ZAF	South Africa
35	CPV	Cape verde	75	JPN	Japan	115	PAN	Panama	155	ZMB	Zambia
36	CRI	Costa Rica	76	KAZ	Kazakhstan	116	PER	Peru	156	ZWE	Zimbabwe
37	CZE	Czech Republic	77	KEN	Kenya	117	PHL	Philippines			
38	DEU	Germany	78	KGZ	Kyrgyz	118	POL	Poland			
39	DJI	Djibouti	79	KHM	Cambodia	119	PRT	Portugal			
40	DNK	Denmark	80	KOR	Korea	120	PRY	Paraguay			
