# Supplementary figures and images for: Air pollution, general government public-health expenditures and income inequality: Empirical analysis based on the spatial Durbin model
Source: PLoS One. 2020 Oct 1;15(10):e0240053. doi: 10.1371/journal.pone.0240053 (PMC7529191; doi:10.1371/journal.pone.0240053)

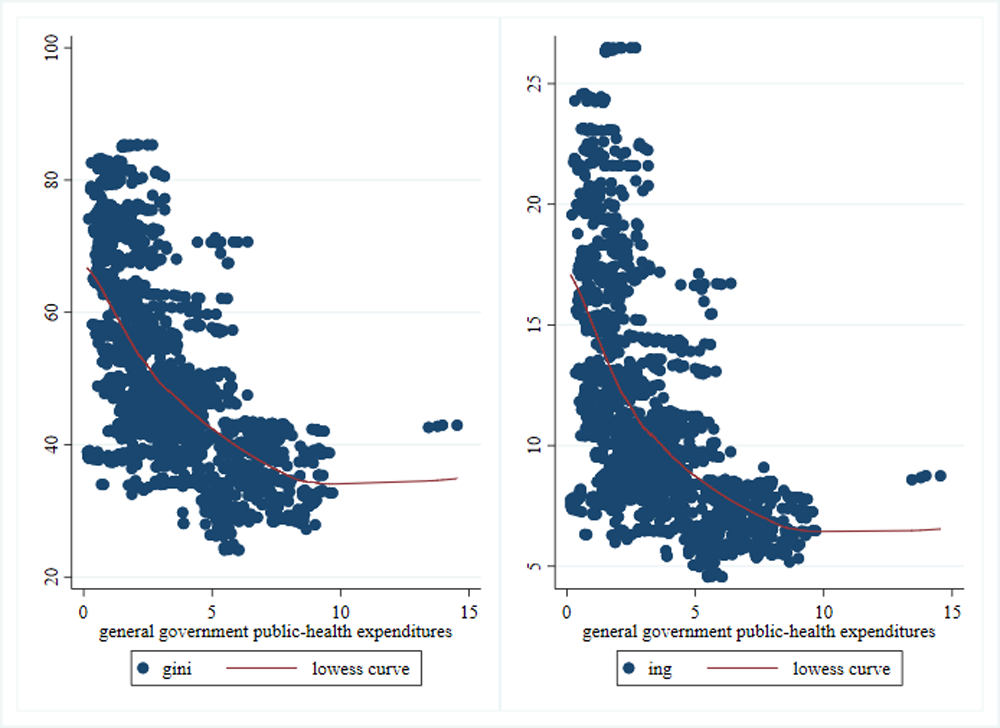

Supplement: S1 Fig — (TIF) [file pone.0240053.s004.tif]

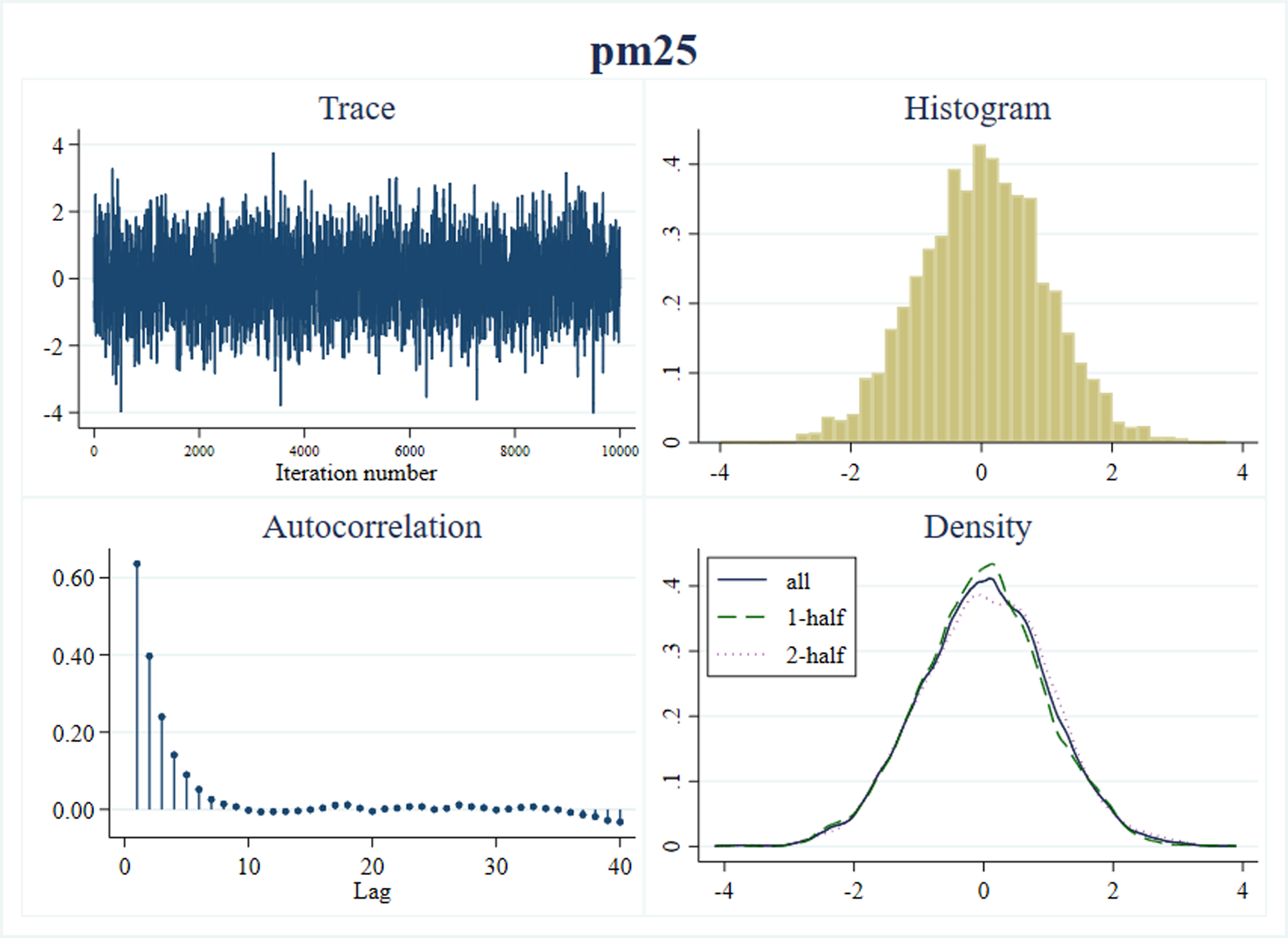

Supplement: S2 Fig — (TIF) [file pone.0240053.s005.tif]

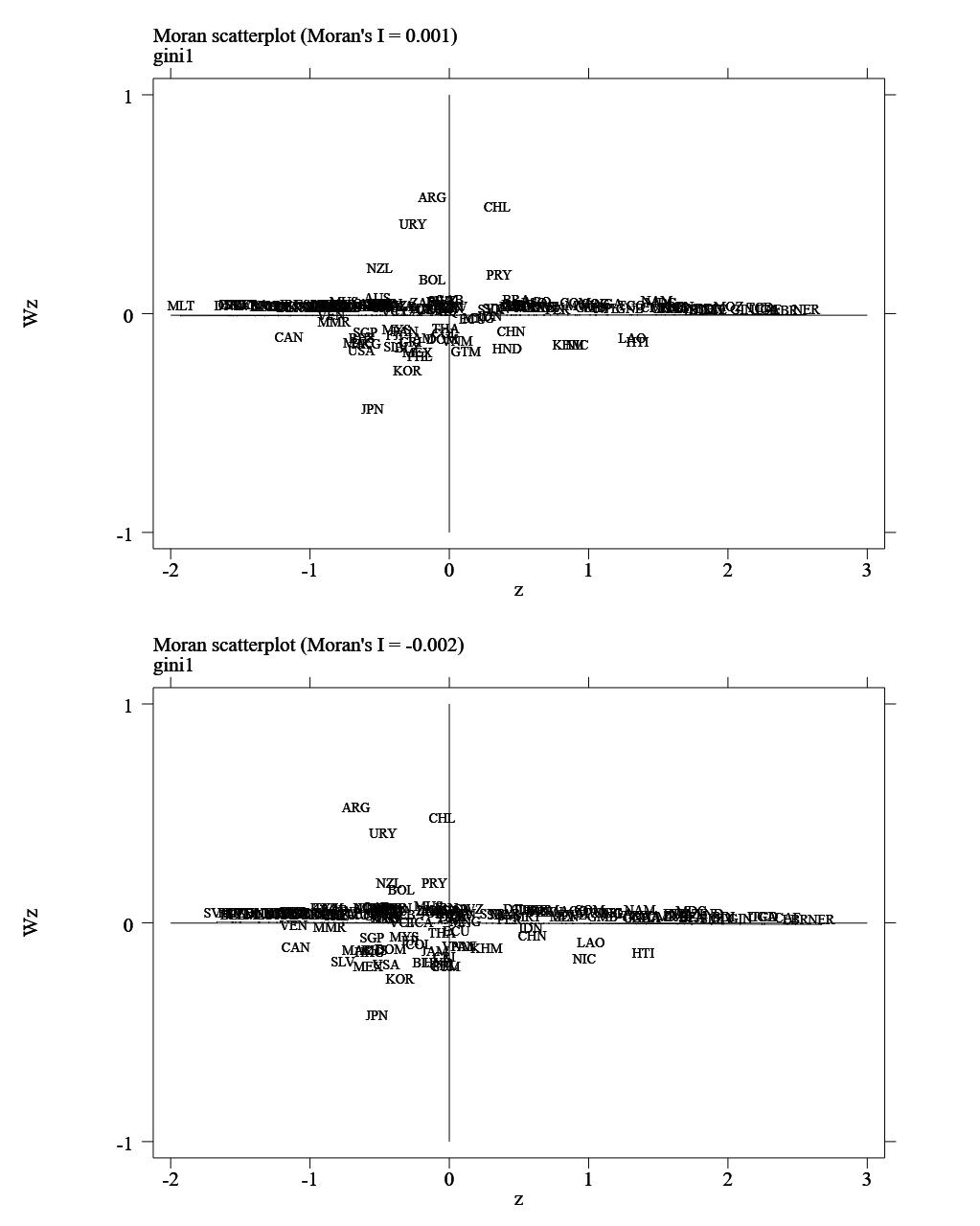

Supplement: S3 Fig — (TIF) [file pone.0240053.s006.tif]

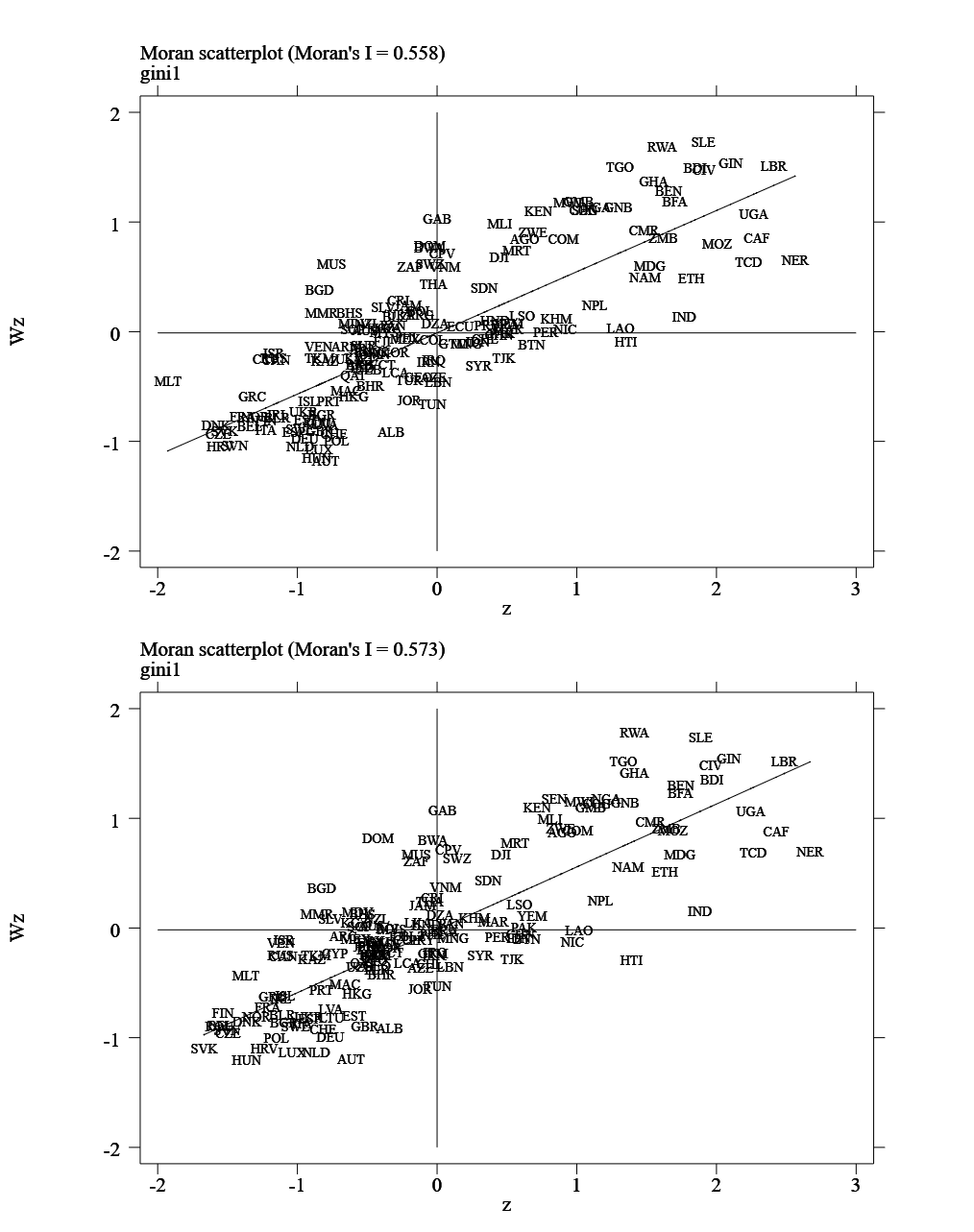

Supplement: S4 Fig — (TIF) [file pone.0240053.s007.tif]

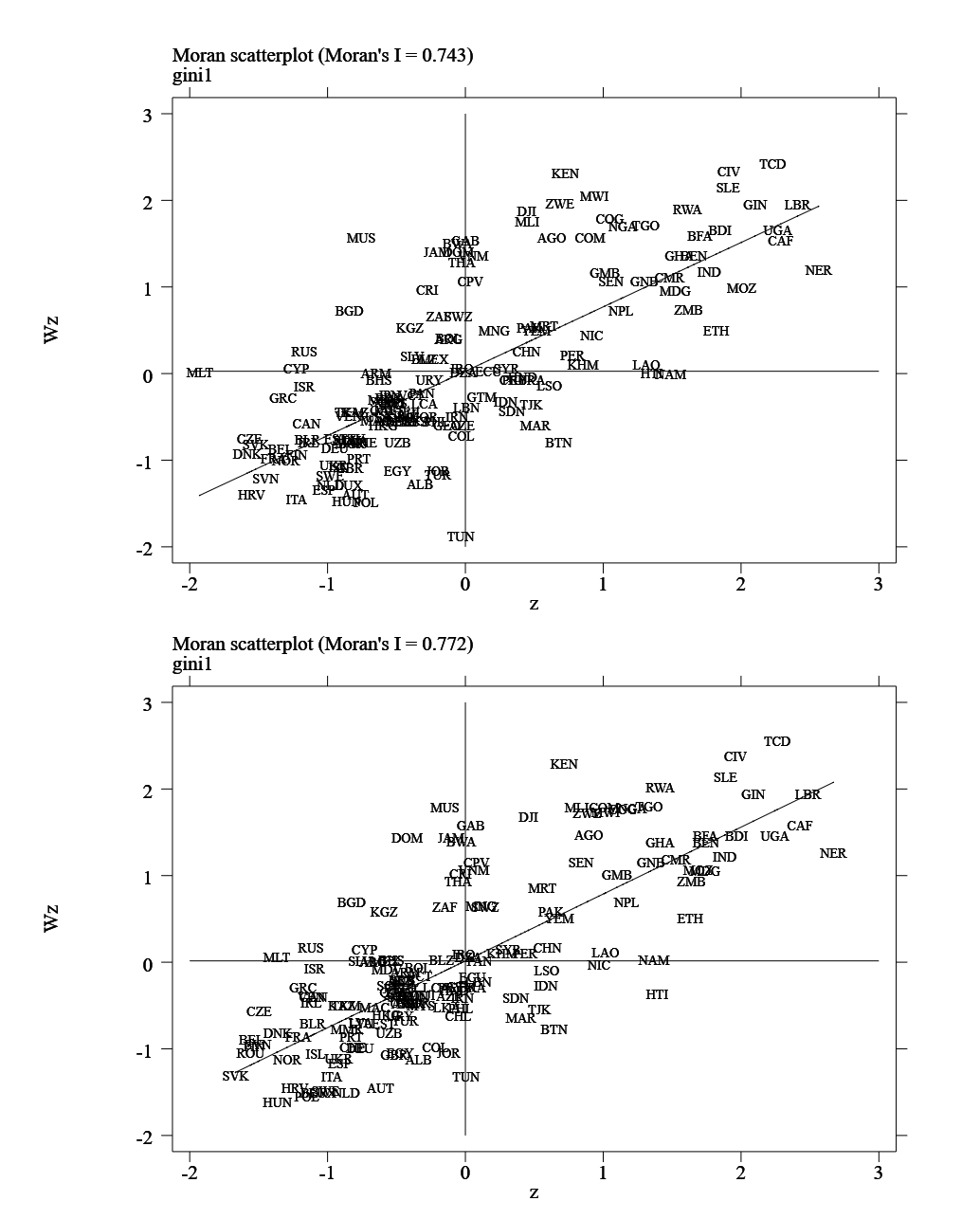

Supplement: S5 Fig — (TIF) [file pone.0240053.s008.tif]
